# Examining the Effects of Caffeine on Isokinetic Strength, Power, and Endurance

**DOI:** 10.3390/jfmk7040071

**Published:** 2022-09-21

**Authors:** Jozo Grgic, Sandro Venier, Pavle Mikulic

**Affiliations:** 1 Institute for Health and Sport, Victoria University, Melbourne, VIC 3011, Australia; 2 Faculty of Kinesiology, University of Zagreb, 10000 Zagreb, Croatia

**Keywords:** ergogenic aid, sports supplements, resistance training

## Abstract

This study examined caffeine’s effects on isokinetic strength, power, and endurance. The sample included 25 young, resistance-trained males. The participants were tested on three occasions, in a control trial (no substance ingestion) and following the ingestion of 6 mg·kg^−1^ of caffeine or placebo. Exercise tests involved isokinetic knee extension and flexion using angular velocities of 60° s^−1^ and 180° s^−1^. Analyzed outcomes included peak torque, average power, and total work. For knee extension at an angular velocity of 60° s^−1^, there were significant differences for: (1) peak torque when comparing caffeine vs. control (Hedges’ *g* = 0.22) and caffeine vs. placebo (*g* = 0.30) and (2) average power when comparing caffeine vs. control (*g* = 0.21) and caffeine vs. placebo (*g* = 0.29). For knee extension at an angular velocity of 180° s^−1^, there were significant differences for: (1) peak torque when comparing caffeine vs. placebo (*g* = 0.26), (2) average power when comparing caffeine vs. control (*g* = 0.36) and caffeine vs. placebo (*g* = 0.43), and (3) total work when comparing caffeine vs. control (*g* = 0.33) and caffeine vs. placebo (*g* = 0.36). Caffeine was not ergogenic for knee flexors in any of the analyzed outcomes. Additionally, there was no significant difference between control and placebo. In summary, caffeine enhances the mechanical output of the knee extensors at lower and higher angular velocities, and these effects are present when compared to placebo ingestion or no substance ingestion (control).

## 1. Introduction

Muscular qualities such as strength, power, and endurance are important for the performance of various sporting tasks as well as activities of daily living [1,2,3,4]. These muscular qualities can be evaluated using different isotonic, isometric, and isokinetic tests [5,6,7]. Isokinetic testing is particularly popular in laboratory-based settings, both among clinical and sporting populations [7]. While each form of testing possesses certain advantages and limitations, several benefits of isokinetic testing should be mentioned. First, in isokinetic tests, there is maximal resistance throughout the range of motion, given that there is no fixed resistance in the “weakest” part of the movement [7]. Second, resistance in this test is also accommodating, which is important from a safety perspective as the accommodating mechanism disengages when the individual performing the test senses pain [7]. Finally, isokinetic testing allows the use and control of different angular velocities and provides several outputs, including:

- Torque—force measured about a joint’s axis of rotation;
- Work—product of muscular force and the distance over which that force was applied;
- Power—amount of work performed in a given amount of time.

Caffeine is a highly popular ergogenic aid, with its performance benefits established in various forms of exercise [8,9,10]. Caffeine’s ergogenic effects are generally explained by its affinity to bind to adenosine receptors [10]. After binding to these receptors, caffeine alleviates sensations of fatigue and reduces perceived exertion, effects which can contribute to improvements in performance [10]. Besides these factors, caffeine has also been shown to increase motor unit recruitment and attenuate exercise-induced reduction in voluntary activation and contractile function [11,12].

Several studies have examined the effects of caffeine on muscular qualities in an isokinetic test. In 2019, a meta-analysis of ten studies compared the effects of caffeine vs. placebo on isokinetic peak torque and observed a small ergogenic effect [13]. While these findings are relevant, several notable limitations are also observed in the current body of evidence. For example, some studies only examined the effects of caffeine on peak torque, whereas caffeine’s effects on other outcomes, such as power and total work remain largely unexplored [14,15]. Additionally, other studies have only focused on one muscle group or used only one angular velocity for the test [15,16,17]. For example, one study [15] provided 6 mg·kg^−1^ to 10 participants who only performed isokinetic knee extension. Timmins and Saunders [17] also provided a caffeine dose of 6 mg·kg^−1^ before evaluating peak torque using only an angular velocity of 60° s^−1^. Even though an ergogenic effect was observed in both studies, these methodological aspects should be considered, as caffeine’s effects may be muscle group-specific, and there is also preliminary evidence supporting angular velocity-specific responses [13,18]. Finally, previous studies on the effects of caffeine on isokinetic strength only compared caffeine to a placebo [14,15,16,17]. The currently available meta-analytical data are also limited to the caffeine vs. placebo comparison [13]. However, researchers have recently started advocating the addition of a no-placebo control trial [19]. Incorporating a control condition is valuable given that, in the practical context, a given individual will either ingest or not ingest caffeine (i.e., using a placebo is not likely to occur). Therefore, comparing the effects of caffeine to a control condition allows for quantifying its actual practical effect. Recent data also suggest that placebo ingestion may be ergogenic compared to a control condition, thus suggesting that a proportion of caffeine’s benefits on exercise performance may be due to placebo effects [20]. However, this aspect has not yet been examined for isokinetic outcomes. 

Given the outlined limitations in the previous studies, our aim was to: (a) explore the effects of caffeine on the mechanical output of the knee extensors and knee flexors at two different angular velocities and (b) examine the effects on peak torque, average power, and total work by comparing the data recorded during the caffeine trial with a placebo and a no-placebo control trial. We hypothesized that only caffeine ingestion would be ergogenic for the knee extensors, regardless of the angular velocity of the test.

## 2. Materials and Methods

### 2.1. Study Design

This study utilized a randomized, crossover, double-blind study design. In total, the participants attended four testing sessions. The first session included familiarization with the exercise tests. The remaining three sessions included isokinetic testing in a control trial (no substance ingestion) or after caffeine or placebo ingestion. In the control condition, the participants came to the laboratory 60 min before exercise but did not ingest any capsules. Caffeine was provided 60 min before exercise, in capsule form, using a 6 mg·kg^−1^ dose. The placebo contained 6 mg·kg^−1^ of dextrose and was also ingested 60 min before exercise. All testing sessions were performed in the morning (between 7:00 and 9:00 a.m.) following an overnight fast. In addition to fasting, the participants were requested to keep their nutritional habits consistent on the days before the three main testing sessions. They were also required not to perform any intense physical activity and limit their caffeine intake after 6 p.m. on the days before the testing sessions. Between the testing sessions, a minimum of three and a maximum of six days were provided.

### 2.2. Participants

We included male resistance-trained participants aged between 18 and 45 years. We defined “resistance-trained” as having at least one year of resistance training experience with a weekly training frequency of at least two times per week (on most weeks). Initially, we included a total of 26 participants. However, one participant felt nauseated following the ingestion of caffeine and could not complete the testing session. Therefore, 25 participants (age: 23 ± 2 years; height: 182 ± 7 cm; weight: 83 ± 11 kg; habitual caffeine intake: 1.0 ± 1.2 mg·kg·day^−1^, range: 0.0 to 4.4 mg·kg·day^−1^) completed all the testing sessions and their data were included in the analysis. The Committee for Scientific Research and Ethics of the Faculty of Kinesiology at the University of Zagreb provided ethical approval for the study [20,21,22,23]. All participants signed informed consent.

### 2.3. Isokinetic Knee Extension and Flexion

The participants first performed a brief (10 min) self-selected warm-up (e.g., light running and stretching), which was standardized for all testing sessions. After the warm-up, isokinetic strength, power, and endurance of the knee extensors and knee flexors were evaluated on an isokinetic dynamometer (System 4 Pro, Biodex Medical Systems, Inc., Shirley, NY, USA). This assessment was performed only for the dominant leg. The participants were first placed in a seated position on a dynamometer. Then, stabilization straps were applied to the trunk, waist, thigh, and shin. The dynamometer’s axis of rotation was set to align with the dominant leg’s lateral femoral epicondyle. Before starting the test, the isokinetic dynamometer was calibrated. We used two angular velocities for the tests, namely, 60° s^−1^ and 180° s^−1^. Testing was first performed for an angular velocity of 60° s^−1^ followed by 180° s^−1^. In both cases, the range of motion of the knee joint was 80°. When starting the test, the participants first performed three warm-up repetitions, which ensured that they got accustomed to the speed of the lever arm. A 30 s rest interval was provided following these three repetitions. Then, the main exercise testing was performed, which involved five maximum knee extensions and knee flexions. We provided the participants with instructions to extend or flex the knee with maximum effort (i.e., “kick” or “pull” as “fast” and “hard” as possible). The outcomes of this test were: peak torque (Nm), average power (W), and total work (J). 

### 2.4. Effectiveness of the Blinding

Caffeine ingestion results in physiological effects such as increased arousal and wakefulness [24]. Due to these effects, some participants may be able to discern caffeine from placebo, thus negating the benefits of a blinded design. To explore the effectiveness of the participants blinding to the caffeine and placebo trials, we utilized the assessment proposed by Saunders et al. [25]. Specifically, we asked the participants to respond to the following question: “Which supplement do you think you have ingested?”, that had three possible answers: (a) “caffeine”, (b) “placebo”, (c) “do not know”. This assessment was performed pre-exercise and post-exercise in the caffeine and placebo trials. 

### 2.5. Statistical Analysis

The performance outcomes (peak torque, average power, and total work) recorded during the three conditions (caffeine, placebo, and control; Table 1) were analyzed using a one-way repeated measures ANOVA. In case of a significant main effect from the ANOVA, pairwise comparisons were performed using a paired *t*-test for caffeine vs. placebo, caffeine vs. control, and placebo vs. control. The statistical significance threshold was initially set at *p* < 0.05. However, to account for multiple comparisons, the significance threshold was adjusted using the Holm correction. This method involved ranking the *p*-values from the three pairwise comparisons for a given outcome from highest to lowest. Adjustments of the statistical significance threshold were performed based on the ranks. For ranks 3, 2, and 1, the statistical significance thresholds were 0.05, 0.025, and 0.017, respectively. Effect sizes and their respective 95% confidence intervals were calculated using Hedges’ *g* for repeated measures. Hedges’ *g* values were interpreted as trivial (<0.20), small (0.20–0.49), medium (0.50–0.79), and large (≥0.80) [26]. We used Bang’s blinding index to examine the effectiveness of the blinding [27]. In this index, values range from −1.0 (opposite guessing) to 1 (complete lack of blinding). These values are presented as the percentage of individuals identifying the trial beyond chance. All analyses were performed using Microsoft Excel (Microsoft Corporation, Redmond, WA, USA).

## 3. Results

### 3.1. Peak Torque

There was a significant main effect for knee extension peak torque at 60° s^−1^ (*p* = 0.002). In pairwise comparisons, a significant difference was found between caffeine vs. control (*p* = 0.012; Hedges’ *g*: 0.22) and caffeine vs. placebo (*p* = 0.004; Hedges’ *g*: 0.30). There was a significant main effect for knee extension peak torque at 180° s^−1^ (*p* = 0.015). In pairwise comparisons, a significant difference was found between caffeine vs. placebo (*p* = 0.006; Hedges’ *g*: 0.26). 

There was a significant main effect for knee flexion peak torque at 60° s^−1^ (*p* = 0.033). However, with the adjusted statistical significance thresholds for multiple comparisons, none of the pairwise comparisons were significant (Table 2). There was no significant main effect for knee flexion peak torque at 180° s^−1^ (*p* = 0.665).

### 3.2. Average Power

There was a significant main effect for knee extension average power at 60° s^−1^ (*p* = 0.003). In pairwise comparisons, a significant difference was found between caffeine vs. control (*p* = 0.012; Hedges’ *g*: 0.21) and caffeine vs. placebo (*p* = 0.008; Hedges’ *g*: 0.29). There was a significant main effect for knee extension average power at 180° s^−1^ (*p* < 0.001). In pairwise comparisons, a significant difference was found between caffeine vs. control (*p* = 0.004; Hedges’ *g*: 0.36) and caffeine vs. placebo (*p* = 0.001; Hedges’ *g*: 0.43). 

There was a significant main effect for knee flexion average power at 60° s^−1^ (*p* = 0.036). However, with the adjusted statistical significance thresholds for multiple comparisons, none of the pairwise comparisons were significant (Table 2). There was a significant main effect for knee flexion average power at 180° s^−1^ (*p* = 0.026). However, with the adjusted statistical significance thresholds for multiple comparisons, none of the pairwise comparisons were significant (Table 2).

### 3.3. Total Work

There was a significant main effect for knee extension total work at 60° s^−1^ (*p* = 0.030). However, with the adjusted statistical significance thresholds for multiple comparisons, none of the pairwise comparisons were significant (Table 2). There was a significant main effect for knee extension total work at 180° s^−1^ (*p* < 0.001). In pairwise comparisons, a significant difference was found between caffeine vs. control (*p* = 0.002; Hedges’ *g*: 0.33) and caffeine vs. placebo (*p* = 0.001; Hedges’ *g*: 0.36). 

There was no significant main effect for knee flexion total work at 60° s^−1^ (*p* = 0.136). There was a significant main effect for knee flexion total work at 180° s^−1^ (*p* = 0.021). However, with the adjusted statistical significance thresholds for multiple comparisons, none of the pairwise comparisons were significant (Table 2).

### 3.4. Effectiveness of the Blinding

In the pre-exercise assessment, 20% of participants identified caffeine, and 44% identified the placebo beyond chance. In the post-exercise evaluation, 28% of participants identified caffeine, and 52% identified the placebo beyond chance.

## 4. Discussion

There are several important findings arising from the results of this study. First, we found that caffeine ingestion enhanced isokinetic strength, power, and endurance in the knee extensors, but not the knee flexors. Second, the ergogenic effects were found at both angular velocities. Third, caffeine was generally ergogenic when compared to placebo and the control conditions and there were no significant differences between placebo and control for any of the analyzed outcomes. In summary, caffeine ingestion enhances knee extensors’ strength, power, and endurance at both lower and higher angular velocities. These effects are generally consistent when caffeine is compared to either control or placebo.

One of the main findings of this study is that caffeine ingestion enhanced isokinetic strength, power, and endurance of the knee extensors, even though such an effect was not observed in the knee flexors. These divergent responses to the effects of caffeine between different muscle groups have also been previously reported [13,18]. For example, a meta-analysis by Warren et al. [18] found an ergogenic effect of caffeine on muscular strength in the knee extensors but not in other muscle groups. These muscle group-specific responses are primarily attributed to differences in their muscle activation [18]. Using the interpolated-twitch electrical stimulation protocol, one study reported that the baseline (i.e., before caffeine ingestion) activation of the knee extensors was at 83%, whereas the activation of elbow flexors was at 97% [28]. Due to the lower baseline activation, it is hypothesized that the increase in motor unit recruitment and muscle contractile properties is most likely to occur in the knee extensors than in other muscle groups [18]. Given that only a handful of studies have directly evaluated the effects of caffeine on knee extensors and flexors, comparison with results from other studies is limited [29,30]. However, one study [29] explored the effects of 5 mg·kg^−1^ of caffeine on peak torque in both muscle groups and reported an ergogenic effect only in the knee extensors—findings that essentially mirror ours. Another study [30] analyzed the same outcomes and reported an absence of an ergogenic effect, even though the data for knee extensors highly favored the caffeine conditions (*p* = 0.053; ES: 0.29). While future studies on the topic would undoubtedly benefit this research area, our results support the notion that caffeine’s ergogenic effects preferably occur in the knee extensors.

A recent meta-analysis explored the effects of caffeine on isokinetic strength at different angular velocities [13]. This analysis reported that caffeine ingestion enhanced strength at angular velocities of 60° s^−1^ and 180° s^−1^, whereas there was no significant difference between the conditions at 30° s^−1^. However, one limitation of these findings is that they are obtained in subgroup analyses of studies that differed in the angular velocities used and other methodological factors, such as caffeine dose and participants’ training status, which could impact the treatment effect [13,14,17,31]. Thus, we directly compared the effects at different angular velocities and generally observed that the effects of caffeine are similar at 60° s^−1^ and 180° s^−1^. Our data also agree with Bazzucchi et al. [14], who reported an ergogenic effect on both angular velocities. However, Tallis et al. [31] reported an ergogenic effect only on 180° s^−1^. As evidence on the effects of caffeine on angular velocities of 60° s^−1^ and 180° s^−1^ is still limited and conflicting, future studies are thus needed to provide further insights on this topic. Future studies should also consider randomizing the order in which different angular velocities are used, given that our and previous studies used a fixed testing order (i.e., 60° s^−1^ followed by 180° s^−1^).

When an ergogenic effect of caffeine was found, it was generally observed compared to placebo and control conditions. The only exception was knee extension peak torque at 180° s^−1^, where an ergogenic effect was found only compared to a placebo. As placebo ingestion was not ergogenic and the blinding was generally effective, we can conclude that caffeine’s effects are largely attributed to physiological effects, such as increased motor unit recruitment [14]. Findings similar to ours are also observed in other exercise modes [32]. For example, one study incorporated caffeine, placebo, and control conditions while using a cycling time trial to evaluate performance [32]. Caffeine ingestion was ergogenic when compared with placebo and control, while there were no significant differences between the placebo and control conditions [32]. Therefore, it seems that the effects of caffeine in a practical context (i.e., caffeine vs. no caffeine) are likely to be of a similar magnitude as observed in the caffeine vs. placebo comparison. When examining the effects of caffeine on exercise, our results are also important from a methodological standpoint as they demonstrate that using only a placebo condition is still a highly valid comparison condition [19]. 

There are several strengths of the present study. Specifically, we evaluated multiple outcomes (i.e., peak torque, average power, and total work) in two muscle groups at lower and higher angular velocities. In the meta-analysis [13] that explored the effects of caffeine on isokinetic strength, the median sample size per included study was 13 participants. We included 25 participants, which is almost two times larger than this median value, highlighting one of the major strengths of the study. We also evaluated the effectiveness of the participants blinding to the caffeine and placebo trials, a procedure that was not used in several other studies on the topic [14,30]. Despite these strengths, certain limitations also need to be considered. While the effectiveness of the blinding was evaluated, its success was high to moderate, given that 20% to 52% of participants identified the conditions beyond random chance. Therefore, a certain proportion of the ergogenic effect of caffeine may be attributed to expectancy [29]. Additionally, as we included only young male resistance-trained participants, these findings may not apply to other population groups (e.g., older adults, females, or untrained individuals). Finally, we found a main effect in some of the analyses for outcomes related to knee flexors. Although a main effect was observed, post hoc tests did not reveal significant differences between specific conditions. Still, it should be mentioned that in most comparisons, the knee extensors’ data highly favored the caffeine conditions. This favoring suggests that caffeine ultimately may produce a practically relevant effect on isokinetic variables, even in this muscle group. Future research is needed to provide further insights into caffeine’s effects on the strength, power, and endurance of the knee flexors.

## 5. Conclusions

We found that caffeine ingestion enhanced isokinetic strength, power, and endurance of the knee extensors. These effects were found at both angular velocities (60° s^−1^ and 180° s^−1^). Caffeine was similarly ergogenic when the data were compared to placebo and control conditions. There were no significant differences between the placebo and control conditions. In summary, caffeine enhances mechanical output of the knee extensors at lower and higher angular velocities, and these effects are present when compared to no substance (control) or placebo ingestion.

## Figures and Tables

**Table 1:** Summary of the isokinetic performance data recorded during the three study conditions.

Exercise Test	Outcome	Caffeine	Placebo	Control
Isokinetic knee extension at 60° s^−1^	Peak torque (Nm)	242 ± 47	228 ± 43	232 ± 41
	Average power (W)	180 ± 40	169 ± 34	172 ± 33
	Total work (J)	1129 ± 242	1060 ± 212	1097 ± 204
Isokinetic knee flexion at 60° s^−1^	Peak torque (Nm)	141 ± 28	135 ± 23	136 ± 21
	Average power (W)	109 ± 24	103 ± 20	104 ± 20
	Total work (J)	708 ± 147	676 ± 132	680 ± 116
Isokinetic knee extension at 180° s^−1^	Peak torque (Nm)	173 ± 25	166 ± 26	168 ± 25
	Average power (W)	335 ± 54	311 ± 55	315 ± 53
	Total work (J)	891 ± 142	838 ± 141	843 ± 143
Isokinetic knee flexion at 180° s^−1^	Peak torque (Nm)	108 ± 33	104 ± 16	103 ± 16
	Average power (W)	204 ± 41	193 ± 38	191 ± 39
	Total work (J)	572 ± 112	545 ± 101	536 ± 100
Data are presented as mean ± standard deviation				

**Table 2:** Summary of the pairwise comparison performed for outcomes with a significant main effect from the one-way repeated measures ANOVA. For each outcome, the three pairwise comparisons, their *p*-values, ranks (ordered from highest to lowest), adjusted significance thresholds, and effect sizes are presented.

Outcome	Pairwise Comparison	*p*-Value	Rank	Adjusted Statistical Significance Threshold	Hedges’ *g* (95% CI)
Knee extension peak torque at 60° s^−1^	Placebo vs. control	0.307	3	0.05	–0.09 (–0.26, 0.07)
	Caffeine vs. control *	0.012	2	0.025	0.22 (0.02, 0.43)
	Caffeine vs. placebo *	0.004	1	0.017	0.30 (0.12, 0.49)
Knee extension peak torque at 180° s^−1^	Placebo vs. control	0.596	3	0.05	–0.07 (–0.26, 0.10)
	Caffeine vs. control	0.032	2	0.025	0.19 (0.01, 0.39)
	Caffeine vs. placebo *	0.006	1	0.017	0.26 (0.09, 0.45)
Knee flexion peak torque at 60° s^−1^	Placebo vs. control	0.802	3	0.05	–0.04 (–0.19, 0.10)
	Caffeine vs. control	0.077	2	0.025	0.19 (–0.03, 0.43)
	Caffeine vs. placebo	0.028	1	0.017	0.23 (0.03, 0.43)
Knee extension average power at 60° s^−1^	Placebo vs. control	0.252	3	0.05	–0.09 (–0.24, 0.06)
	Caffeine vs. control *	0.012	2	0.025	0.21 (0.04, 0.39)
	Caffeine vs. placebo *	0.008	1	0.017	0.29 (0.07, 0.52)
Knee extension average power at 180° s^−1^	Placebo vs. control	0.450	3	0.05	–0.07 (–0.26, 0.12)
	Caffeine vs. control *	0.004	2	0.025	0.36 (0.11, 0.63)
	Caffeine vs. placebo *	0.001	1	0.017	0.43 (0.21, 0.66)
Knee flexion average power at 60° s^−1^	Placebo vs. control	0.974	3	0.05	–0.05 (–0.25, 0.15)
	Caffeine vs. control	0.045	2	0.025	0.24 (0.00, 0.48)
	Caffeine vs. placebo	0.035	1	0.017	0.26 (0.03, 0.50)
Knee flexion average power at 180° s^−1^	Placebo vs. control	0.664	3	0.05	0.05 (0.14, 0.25)
	Caffeine vs. placebo	0.051	2	0.025	0.27 (–0.01, 0.56)
	Caffeine vs. control	0.029	1	0.017	0.31 (0.03, 0.61)
Knee extension total work at 60° s^−1^	Caffeine vs. control	0.174	3	0.05	0.14 (–0.06, 0.34)
	Placebo vs. control	0.135	2	0.025	–0.17 (–0.41, 0.06)
	Caffeine vs. placebo	0.023	1	0.017	0.29 (0.03, 0.57)
Knee extension total work at 180° s^−1^	Placebo vs. control	0.680	3	0.05	–0.03 (–0.18, 0.11)
	Caffeine vs. control *	0.002	2	0.025	0.33 (0.11, 0.55)
	Caffeine vs. placebo *	0.001	1	0.017	0.36 (0.15, 0.59)
Knee flexion total work at 180° s^−1^	Placebo vs. control	0.279	3	0.05	0.09 (–0.08, 0.26)
	Caffeine vs. placebo	0.094	2	0.025	0.24 (–0.04, 0.54)
	Caffeine vs. control	0.0175	1	0.017	0.33 (0.06, 0.62)

CI: confidence interval; * denotes statistically significant differences even after adjusting the significance threshold using the Holm–Bonferroni correction. Note: as there was no significant main effect from the one-way repeated measures ANOVA for knee flexion peak torque at 180° s^−1^ and knee flexion total work at 60° s^−1^, pairwise comparisons for these outcomes were not performed.

## Data Availability

Data are available upon request.
